# Travel-related Dengue Virus Infection, the Netherlands, 2006–2007

**DOI:** 10.3201/eid1705.101125

**Published:** 2011-05

**Authors:** Gijs G.G. Baaten, Gerard J.B. Sonder, Hans L. Zaaijer, Tom van Gool, Joan A.P.C.M. Kint, Anneke van den Hoek

**Affiliations:** Author affiliations: Public Health Service, Amsterdam, the Netherlands (G.G.G. Baaten, G.J.B. Sonder, J.A.P.C.M. Kint, A. van den Hoek);; Academic Medical Centre, Amsterdam (G.G.G. Baaten, G.J.B. Sonder, H.L. Zaaijer, T. van Gool, A. van den Hoek);; National Coordination Centre for Traveller’s Health Advice, Amsterdam (G.G.G. Baaten, G.J.B. Sonder)

**Keywords:** Dengue, epidemiology, risk factors, prospective study, viruses, the Netherlands, vector-borne infections, travelers, viruses, research

## Abstract

To assess the incidence of and risk factors for clinical and subclinical dengue virus (DENV) infection, we prospectively studied 1,207 adult short-term travelers from the Netherlands to dengue-endemic areas. Participants donated blood samples for serologic testing before and after travel. Blood samples were tested for antibodies against DENV. Seroconversion occurred in 14 (1.2%) travelers at risk. The incidence rate was 14.6 per 1,000 person-months. The incidence rate was significantly higher for travel during the rainy months. Dengue-like illness occurred in 5 of the 14 travelers who seroconverted. Seroconversion was significantly related to fever, retro-orbital pain, myalgia, arthralgia, and skin rash. The risk for DENV infection for short-term travelers to dengue-endemic areas is substantial. The incidence rate for this study is comparable with that in 2 other serology-based prospective studies conducted in the 1990s.

Dengue virus (DENV) is a flavivirus transmitted through the bite of an infected *Aedes* spp. mosquito. The disease is endemic to tropical and subtropical regions and each year affects ≈100 million persons worldwide. Most cases are reported from Southeast Asia and Central and South America (*1**,**2*).

The incubation period for DENV infection is 3–14 days. Manifestations are nonspecific and range from asymptomatic to severe (*3**,**4*). Most infections are benign, with few deaths. In 2%–3% of patients, dengue hemorrhagic fever develops (*5*).

DENV has 4 distinct serotypes. Primary infection with 1 serotype confers lifelong immunity to that serotype but increases the risk for severe dengue illness after secondary infection with another serotype (*6*). No specific therapy exists for dengue.

During the past few decades, dengue has emerged in tropical and subtropical countries worldwide (*1**,**7*). In addition, the number of reported symptomatic DENV infections among international travelers has increased (*5**,**8**,**9*). This increase may reflect increased incidence of dengue among travelers, increased number of travelers to areas in which dengue is endemic, increased awareness of the disease among physicians, or a combination of these factors.

However, valid research on travelers’ risk for DENV infection is scarce. To our knowledge, only 2 prospective studies, performed in the 1990s (*10**,**11*), have been conducted. Most other epidemiologic studies are retrospective and focus on symptomatic patients who seek care at a clinical site, thus disregarding the nonspecific nature of the infection and the increased number of travelers to dengue-endemic areas (*5**,**8**,**9**,**12**,**13*).

We prospectively estimated the prevalence and incidence of DENV infection and its risk factors. Our findings are based on serologic testing in a cohort of short-term travelers from the Netherlands to dengue-endemic areas.

## Methods

### Study Population

We prospectively studied persons attending the travel clinic of the Public Health Service Amsterdam during October 1, 2006–September 30, 2007. All persons >18 years of age were eligible if they planned to travel for 1–13 weeks to >1 countries to which dengue is endemic. Dengue endemicity was based on information from The Global Infectious Diseases and Epidemiology Online Network (*14*).

All participants were seen by a medical doctor or nurse who specialized in travel medicine. They received vaccinations, a prescription for antimalarial chemoprophylaxis if required, and oral and written information about how to avoid acquiring travel-related diseases, including mosquito-borne infections, according to Dutch National Guidelines on Traveler’s Health Advice (*15*).

### Survey Methods

Before departure (just before they received the required vaccinations) and 2–6 weeks after return, participants donated venous blood samples for serologic testing. Before departure,they completed a standard questionnaire to collect data on sociodemographic factors, including travel history, vaccination status, and the purpose of travel (tourism, work/education, or visits to friends and/or relatives).

Participants were given a digital thermometer and asked to take their temperature (axillary or rectally) when feeling feverish. They kept a structured travel and posttravel diary to record symptoms and signs of dengue illness (fever, myalgia, arthralgia, headache, retro-orbital pain, skin rash, signs and symptoms of gastrointestinal disease), use of insect repellent containing N,N-diethyl-meta-toluamide (DEET), and use of mosquito netting. Participants were asked to report both the presence and absence of symptoms and preventive measures on a daily basis; after travel, the diary was checked by a registered nurse in the participant’s presence.

To encompass the incubation period of DENV infection, we used data from the third day after arrival at the travel destination until 2 weeks after return from travel. With respect to symptomatic outcome, we used the term travel-related to refer to this period.

Destinations were grouped into regions by using the classification of the United Nations Development Agency (*16*). Travel duration was recorded as the total days spent in areas meeting the inclusion criteria.

The Medical Ethics Committee of the Academic Medical Centre Amsterdam approved the study protocol. Participants were included only if they provided informed and written consent.

### Laboratory Methods

Blood samples were immediately stored at 6°C, then centrifuged and frozen at –80°C within 24 h after collection. After all study participants had returned, all posttravel serum samples were thawed and tested for immunoglobulin (Ig) M antibodies to DENV antigen by using an IgM-capture ELISA and for IgG antibodies to DENV antigen serotypes 1, 2, 3, and 4 by using an indirect ELISA (Panbio Diagnostics, Brisbane, Queensland,Australia) according to the manufacturer’s instructions. For participants whose posttravel sample yielded positive test results for anti-DENV IgM or IgG, pretravel samples were also tested for anti-DENV IgM or IgG, respectively.

The ELISA for anti-DENV IgM has a sensitivity of 87%–100% and a specificity of 96% (*17**–**19*). The ELISA for anti-DENV IgG has a sensitivity of 99%–100% and a specificity of 96%–98% (*17**,**20*). These test characteristics concern the use of paired serum samples.

Serology suggestive for previous DENV infection was defined as an anti-DENV IgG >11 Panbio units in both the pretravel and the posttravel sample. The posttravel-to-pretravel anti-DENV IgG ratio was <4:1.

We defined seroconversion indicating recent DENV infection. Criteria were as follows: 1) posttravel anti-DENV IgM >11 Panbio units with pretravel anti-DENV IgM <11 Panbio units and anti-DENV IgG <11 Panbio units; and/or 2) posttravel anti-DENV IgG >11 Panbio units and pretravel anti-DENV IgG <11 Panbio units, with a posttravel-to-pretravel ratio of >4:1; and/or 3) anti-DENV IgG >11 Panbio units in both the pretravel and posttravel sample, with a posttravel-to-pretravel ratio of >4:1 (*21*).

### Data Analysis

To calculate risk factors for previous DENV infection, we used SPSS for Windows version 17.0 (SPSS Inc., Chicago, IL, USA) to obtain prevalence rates, univariate and multivariate prevalence rate ratios, and 95% confidence intervals (CIs) by using logistic regression modeling. All variables with an overall p value <0.05 in univariate analysis were included in multivariate analysis. Statistical interactions between variables were checked for all variables. If significant (p<0.05), they were included in multivariate analysis.

We calculated attack rates of recent DENV infection per 100 travelers by dividing the number of seroconversions by the total number of participants. Incidence rates (IR) per 1,000 person-months were calculated by dividing the number of seroconversions by the total number of months in which participants were at risk for DENV infection. To calculate risk factors for recent DENV infection, incidence rate ratios (IRR) were obtained as conditional maximum-likelihood estimates of the rate ratio, with mid-P exact test, 95% CIs, and p values. We quantified the application of a protective measure against mosquito bites by dividing the number of days the measure was applied by the number of travel days; it was dichotomized by using the mean of this proportion in the total study population as the cutoff.

Travel during the rainy months was assessed by using the midtravel dates. The rainy months per destination were defined as months with an average total precipitation of >100 mm, based on World Weather Information Service data from the United Nations’ World Meteorological Organisation (*22*).

We used χ^2^ tests to evaluate the predictive value of travel-related signs and symptoms for recent DENV infection. Fever was defined as a body temperature >38°C. Dengue-like illness was defined as any episode of fever accompanied by >1 of the following: myalgia, arthralgia, headache, retro-orbital pain, or skin rash. A p value <0.05 was considered significant.

## Results

### Study Population

The study began with 1,276 participants who intended to travel to dengue-endemic areas. Of these, 69 (5.4%) were excluded: 33 were lost to follow-up; 23 had their travel arrangements cancelled; 7 did not supply the posttravel blood donation; 3 changed travel plans such that inclusion criteria were not met; 2 did not complete the structured diary; and for 1, the posttravel sample was lost.

Most of the remaining 1,207 participants were persons of Dutch origin who were traveling for tourism and previously had traveled to dengue-endemic areas (Tables 1, 2). The median travel duration was 21 days, with an interquartile range (IQR) of 16–28. The median interval between return from travel and posttravel blood donation was 25 days (IQR 21–29).

**Table 1:** Characteristics of 1,207 persons who traveled from the Netherlands to dengue-endemic countries and who attended a travel clinic for pretravel health advice, October 1, 2006–September 30, 2007*

Characteristic	Total, no. (%)	Previous DENV			Univariate analysis			Multivariate analysis	
		No.	PR, %		PRR (95% CI)	p value		PRR (95% CI)	p value
No. participants	1,207 (100)	78	6.5		NA				
Sex									
M	521 (43)	29	5.6		1				
F	686 (57)	49	7.1		1.3 (0.81–2.1)	0.27			
Median age, y, IQR	38, 29–51	49, 35–59			1.04 (1.02–1.06)	<0.0001		1.03 (1.01–1.05)	0.003
Country of birth									
Non–dengue-endemic country	1,118 (93)	40	3.6		1			1	
Dengue-endemic country	89 (7)	38	43		20.1 (11.9–34.0)	<0.0001		14.4 (8.1–25.6)	<0.0001
No. times previously traveled to a dengue-endemic country						<0.0001			0.004
0–1	447 (37)	5	1.1		1			1	
2–5	477 (40)	35	7.3		7.0 (2.7–18.0)	<0.0001		5.0 (1.8–13.9)	0.002
>6	283 (23)	38	13.4		13.7 (5.3–35.3)	<0.0001		6.4 (2.1–19.2)	0.001
Previous travel destinations									
Not Asia	537	31	5.8		1				
Asia	670 (56)	47	7.0		1.2 (0.77–2.0)	0.38			
Not Latin America	661	22	3.3		1			1	
Latin America	546 (45)	56	10.3		3.3 (2.0–5.5)	<0.0001		1.6 (0.85–2.9)	0.15
Not Africa	679	41	6.0		1				
Africa	528 (48)	37	7.0		1.2 (0.74–1.9)	0.50			
Primary purpose of current travel						<0.0001			0.001
Tourism	1,032 (86)	52	5.0		1			1	
Work or education	99 (8)	8	8.1		1.7 (0.76–3.6)	0.20		1.3 (0.52–3.3)	0.57
Visit with friends and/or relatives	76 (6)	18	23.7		5.8 (3.2–10.6)	<0.0001		4.1 (2.0–8.5)	<0.0001
Vaccination status									
Not vaccinated for previous travel	888 (74)	46	5.2		1			1	
Vaccinated for previous travel	319 (26)	32	10.0		2.0 (1.3–3.3)	0.003		1.0 (0.56–1.78)	0.99

*DENV, dengue virus; PR, prevalence rate; PRR, prevalence rate ratio; CI, confidence interval; NA, not applicable; IQR, interquartile range.

**Table 2:** Characteristics of travelers with serologic evidence of recent dengue virus infection from a cohort of 1,207 persons who traveled from the Netherlands to developing countries, October 1, 2006–September 30, 2007*

Traveler no.: age, y/sex	Country of birth	Destination country (region)	Previous times traveled to developing country	Travel-related signs and symptoms	Dengue-like illness	Vaccinated against yellow fever	IgM-ratio†	IgG-ratio†
1: 33/M	The Netherlands	Philippines (Asia)	2–5	Fever, myalgia, headache, retro-orbital pain, diarrhea	Yes	Never	NA	17.65
2: 62/M	Germany	Indonesia (Asia)	0	Skin rash, diarrhea	No	Never	51.79	38.9
3: 64/M	The Netherlands	Sri Lanka (Asia)	>10	Fever, myalgia, arthralgia, head-ache, retro-orbital pain, diarrhea	Yes	In 1992	NA	28.3
4: 41/M	The Netherlands	Cambodia, Thailand, Malaysia, Philippines (Asia)	>10	None	No	Never	NA	46.27
5: 36/F	The Netherlands	Thailand, Singapore (Asia)	6–10	Diarrhea	No	Never	3.34	NA
6: 37/F	The Netherlands	Indonesia (Asia)	0	Fever, myalgia, headache, retro-orbital pain, skin rash	Yes	Never	48.66	142.25
7: 28/M	The Netherlands	Vietnam (Asia)	2–5	None	No	Never	2.78	NA
8: 27/M	The Netherlands	India, Nepal (Asia)	2–5	Diarrhea	No	Never	5.11	NA
9: 68/M	The Netherlands	Surinam (Latin America)	1	None	No	In 2000	NA	8.91
10: 26/F	The Netherlands	Argentina, Bolivia, Brazil, Chili, Uruguay (Latin America)	0	Fever, myalgia, arthralgia, headache, skin rash, diarrhea	Yes	For this trip	NA	26.67
11: 23/F	The Netherlands	Surinam (Latin America)	1	Skin rash, diarrhea	No	For this trip	NA	72.82
12: 54/F	The Netherlands	Gambia (Africa)	2–5	Fever, myalgia, arthralgia, head-ache, retro-orbital pain, diarrhea	Yes	For this trip	NA	26.87
13: 27/M	The Netherlands	Mali, Burkina Faso (Africa)	2–5	Skin rash, diarrhea	No	In 2000	18.15	NA
14: 52/M	The Netherlands	Madagascar (Africa)	6–10	Diarrhea	No	In 1992	8.82	NA

*Ig, immunoglobulin; NA, ratio not available. †Posttravel to pretravel. If the posttravel sample test results were negative for a type of anti-DENV, the pretravel serum sample was not tested for that type of antibody.

Twenty-six percent of travelers had received yellow fever vaccination for a previous journey, and 23% received a yellow fever vaccination for the current trip, just after blood donation (not shown in Table 1). Only 4 (0.3%) participants had ever received Japanese encephalitis vaccination, all for a previous journey. No one had ever received vaccination against tick-borne encephalitis.

### Serologic Results Suggestive for Previous DENV Infection

Serologic results suggestive of previous DENV infection were found for 78 (6.5%; 95% CI 5.2%–8.0%) participants (Table 1). Previous DENV infection was positively correlated with increasing age. The rate was significantly higher for travelers born in a dengue-endemic country, for those who traveled frequently to dengue-endemic countries (all 78 reported >1 previous trips to such a country), and for those whose purpose was to visit friends or relatives. In multivariate analysis, previous DENV infection was not related to yellow fever vaccination for previous travel because it was confounded by previous trips to a developing country. We found no interactions indicating effect modification.

### Serology Suggestive of Recent Dengue Infection

Fourteen participants (1.2%; 95% CI 0.66%–1.9%) had recent DENV infection, of whom 5 seroconverted only for IgM; 7 seroconverted only for IgG, with a posttravel-to-pretravel ratio of >4:1; and 2 seroconverted for both IgM and IgG (Table 2). Thus, the attack rate for recent DENV infection was 1.2% (95% CI 0.66%–1.9%). The incidence rate was 14.6 per 1,000 person-months (95% CI 8.3–23.9) (Table 3).

**Table 3:** Attack rates and incidence rates of seroconversions in antibody levels for dengue virus in 1,207 persons who traveled from the Netherlands to dengue-endemic countries, October 1, 2006–September 30, 2007*

Region	No. (%) persons at risk	No. seroconversions	Person-months of travel	Attack rate, % (95% CI)	Incidence rate, %† (95% CI)
All	1,207 (100)	14	959.2	1.2 (0.66–1.9)	14.6 (8.3–23.9)
Asia	569 (47)	8	474.3	1.4 (0.66–2.7)	16.9 (7.8–32.0)
Southeast Asia	375 (31)	6	324.0	1.6 (0.82–3.7)	18.5 (9.4–42.7)
South Asia	147 (12)	2	116.4	1.4 (0.034–3.3)	17.2 (0.43–42.4)
East Asia	98 (8)	0	86.2	0	0
Latin America	340 (28)	3	261.3	0.9 (0.23–2.4)	11.5 (2.9–31.3)
South America	219 (18)	3	184.5	1.4 (0.35–3.7)	16.3 (4.1–44.3)
Central America and Caribbean	135 (11)	0	96.0	0	0
Africa	298 (25)	3	223.5	1.0 (0.26–2.7)	13.4 (3.4–36.5)
West and Middle Africa	113 (9)	2	75.4	1.8 (0.30–5.7)	26.5 (4.5–87.6)
East Africa	140 (12)	1	109.4	0.7 (0.036–3.5)	9.1 (0.46–45.1)
Southern Africa	67 (6)	0	57.0	0	0

*CI, confidence interval; NA, not applicable. †Per 1,000 person-months.

All 14 persons with recent infection were born in non–dengue-endemic countries; 9 were male, and 11 had traveled previously to a dengue-endemic country (Table 3). The median age was 36 years (IQR 27–56 years). The primary purpose of travel was tourism (12 persons) or work/education (2 persons). The median travel duration was 24 days (IQR 19–30).

The incidence rate was significantly higher for travel during the rainy months (23.6 vs. 7.5/1,000 person-months; IRR 3.2, 95% CI 1.01–11.6; p = 0.048). Travel during the rainy months was not related to use of insect repellent containing DEET or use of a mosquito net (odds ratio 1.0; p>0.05).

The incidence rate appeared to be higher for male travelers (22.3 vs. 9.0; IRR 2.5, 95% CI 0.83–8.2; p = 0.11), persons who traveled for work or education (23.6 vs. 13.7; IRR 1.7, 95% CI 0.26–6.8; p = 0.47), and those who used insect repellent containing DEET <45% of travel days (18.6 vs. 9.5; IRR 2.0, 95% CI 0.63–7.2; p = 0.26), but differences were not significant. With regard to age, previous travel to a dengue-endemic country, yellow fever vaccination for the current trip, and use of a mosquito net, the IRRs equaled 1.0 (p>0.05).

Recent DENV infection was positively correlated with fever, retro-orbital pain, myalgia, arthralgia, and skin rash (Table 4). Dengue-like illness was reported by 5 (36%; 95% CI 14%–62%) of 14 participants. Evidence for recent DENV infection was 7.3× more prevalent among those with than without dengue-like illness. No traveler reported symptoms signs or suggestive of dengue hemorrhagic fever.

**Table 4:** Signs and symptoms among travelers with seroconversion in antibody level for dengue virus in 1,207 persons who traveled from the Netherlands to dengue-endemic countries, October 1, 2006–September 30, 2007*

Sign or symptom	Total no.	Recent DENV, no. (PR, %)	PRR (95% CI)	p value
Fever, temperature >38°C	113	5 (4.4)	5.6 (1.8–17.0)	**0.002**
No fever	1,094	9 (0.8)	1	
Headache	398	5 (1.3)	1.1 (0.38–3.4)	0.83
No headache	809	9 (1.1)	1	
Retro-orbital pain	100	4 (4.0)	4.6 (1.4–14.8)	**0.011**
No retro-orbital pain	1,107	10 (0.9)	1	
Myalgia	166	5 (3.0)	3.6 (1.2–10.8)	**0.024**
No myalgia	1,041	9 (0.9)	1	
Arthralgia	72	3 (4.2)	4.4 (1.2–16.3)	**0.025**
No arthralgia	1,135	11 (1.0)	1	
Rash	105	5 (4.8)	6.1 (2.0–18.5)	**0.001**
No rash	1,102	9 (0.8)	1	
Diarrhea	581	10 (1.7)	2.7 (0.85–8.7)	0.092
No diarrhea	626	4 (0.6)	1	
Dengue-like illness†	89	5 (5.6)	7.3 (2.4–22.4)	**<0.0001**
No dengue-like illness	1,118	9 (0.8)	1	

*DENV, dengue virus infection; PR, prevalence rate; PRR, prevalence rate ratio; CI, confidence intervals. **Boldface** indicates significance (p<0.5). †Fever (temperature >38°C) with >1 of the following: myalgia, arthralgia, headache, retro-orbital pain, or skin rash.

## Discussion

In this prospective study, the serology-based attack rate and incidence rate for recent DENV infection in short-term travelers to areas in which dengue is endemic were substantial: 1.2% and 14.6 per 1,000 person-months, respectively. Although most seroconversions occurred in travelers to Southeast and southern Asia, 3 occurred in travelers to sub-Saharan Africa.

The risk for recent infection was significantly related to travel during the rainy months, as others have reported (*10*). The use of insect repellent containing DEET appeared to be protective, although not significantly so. The presence of fever, retro-orbital pain, myalgia, arthralgia, and skin rash all had predictive value for recent infection. Dengue-like illness was the strongest predictor. Nevertheless, 64% of infections were asymptomatic.

For as many as 6.5% of travelers, serology results suggested previous DENV infection. This rate was strongly related to birth in a dengue-endemic country and a history of frequent travel to such countries. Increasing age and the travel purpose of visiting friends or relatives were also predictive for previous DENV infection, as described (*5*).

Studies based on febrile travelers returning from the tropics have reported dengue fever in ≈2% of those who sought medical help (*12**,**23*). Some studies have suggested that this proportion has increased in the past 2 decades (*3*,*8**,**9**,**24*); others showed no significant increase (*25*).

All these studies were retrospective. They were based on persons who sought medical attention after travel and thereby missed persons who never had symptoms, whose symptoms resolved during travel, or who never consulted a doctor. These studies were influenced by referral bias and relied only on posttravel serologic confirmation. They could not compare characteristics between patients and persons who remained well, and the studies lacked valid denominator data to determine absolute risk. Interpreting their findings is therefore difficult.

The best methodologic approach for estimating incidence rates of clinical and subclinical travel-related DENV infections is to follow a cohort of travelers prospectively (*26*). We found only 2 such studies. In 1 study, 6.7% of 104 travelers from Israel who spent on average 6.1 months (range 3–16 months) in Southeast Asia, South America, or Africa seroconverted (*11*), yielding an incidence rate of 11.0 per 1,000 person-months. This incidence rate is comparable to the rate found in our study, although our study differed in travelers’ destination and duration of exposure and in test characteristics.

In the other prospective study, performed during 1991–1992, the seroconversion rate for 447 travelers from the Netherlands who spent a median of 4 weeks in Asia was 3.6%, resulting in an incidence rate of 36.9 per 1,000 person-months (*10*). In our study, the seroconversion and incidence rates in travelers to Southeast Asia were lower: 1.6% and 18.5 per 1,000 person-months, respectively. Our different findings may reflect different test characteristics and differences in exposure and risk behavior related to factors such as travel destination, year of exposure, and measures taken to prevent mosquito bites.

The incidence rate for persons in our study who traveled to sub-Saharan Africa appeared to be substantial (13.4/1,000 person-months), although the 95% CI was wide (3.4–36.5), and cross-reactions with other flaviviruses or previous yellow fever vaccination cannot be excluded. However, cross-reactions with yellow fever vaccination are unlikely because the travelers to Africa who seroconverted for anti-DENV either did not receive a yellow fever vaccination during the study period (2 travelers) or had dengue-like illness (1) (Table 3). These travelers visited Senegal and the Gambia, Mali and Burkina Faso, and Madagascar. All these countries reported dengue cases during the study period (*14**,**27**,**28*). According to data from the GeoSentinel Network (*12*) and the TropNetEurope Network (*5*), only a small proportion (5%–8%) of clinical dengue cases in travelers are acquired in Africa. However, distributions yielded by these data reflect not only global dengue activity but also the popularity of tourist destinations and physicians’ expectations about dengue endemicity and their inclination to test for the disease. Dengue endemicity in Africa may have changed, and the risk for travelers may thus be higher than expected (*27*). More studies are needed.

The ratio of apparent to unapparent infection in our study was 1:1.8. The 2 other prospective studies reported ratios of 1:0.75 (*11*) and 1:3 (*10*). Variations in these ratios may reflect variation in strain virulence, the influence of initial viral load, or host factors such as susceptibility.

Our study has some limitations. First, our sample size was too small to yield a precise estimate of disease incidence per region or per destination. For example, travelers to Southeast Asia are considered to be at higher risk than travelers to other regions (*5**,**12*). This finding could not be confirmed in our study. Second, selection bias may have occurred. Although participants in our study are comparable to the average traveler (*29*), they were all seeking pretravel health advice. Thus, they perhaps had a higher than average health awareness, particularly after receiving oral and written travel advice, learning about the study, and agreeing to participate. Nevertheless, their average use of insect repellent containing DEET was only 45% of travel days, and 17% of participants used no repellent at all. Third, the risk for travel-related DENV infection also can depend on endemicity and on outbreaks in a particular country during a particular time of travel. Also, among travelers, DENV transmission has annual oscillations (*12**,**30*). Furthermore, sometimes epidemics occur in tourist areas, other times in areas never reached by tourists. Although 2006–2007 were not years with high endemicity for most dengue-endemic countries, the exact contribution of these factors on our findings is unknown.

A final limitation is that, although the serologic tests used are highly sensitive and specific, false-negative and false-positive test results may have occurred. For example, 7 travelers seroconverted only for anti-DENV IgG and not for anti-DENV IgM. Although this may be because of false-positive IgG or false-negative IgM test results, these results may also suggest secondary infection. Unfortunately, no test characteristics are available that apply exactly to our study design; the best available test characteristics are probably those from studies that used paired serum samples (*6**,**17**,**18**,**20*). With test specificity of <96%, >4% of positive samples actually give false-positive results. Subclinical infections with flaviviruses other than DENV may account for some of the seroconversions (*31*). Also, cross-reactions can affect the interpretation of test results in travelers who have received a flavivirus vaccine. In 1 study, 2 (15%) of 13 persons vaccinated against yellow fever had positive test results for DENV-IgG 1–2 months after the vaccination (*31*). In our study, however, recent and previous DENV infections were not related to yellow fever vaccination. False-positive test results can also occur just by chance. Because the serologic tests used were validated in a clinical environment among patients suspected to have dengue, their positive predictive value will be lower when applied as a screening tool in a more general sample of travelers in which disease occurrence is lower.

Unfortunately, no true standard exists for confirming or ruling out DENV infections. Even a plaque-reduction neutralization test can cross-react (*32*), and PCR is useful only in the very early stage of dengue disease (*33*).

In conclusion, although valid data regarding the actual frequency of DENV infection in international travelers is elusive, our prospective study confirms that the incidence rate for recent DENV infection in short-term travelers to areas in which dengue is endemic is substantial. We found no differences in incidence rate compared with other serology-based prospective cohort studies performed in the 1990s. Thus, the increase in annually reported dengue cases from network studies is possibly related to the increase in international travel and the expansion of DENV and its vector to new areas rather than to an increase in DENV transmission. Until an effective and safe vaccine that provides effective, long-term immunity against all 4 serotypes of DENV has been developed, the only useful preventive measure for travelers to areas where dengue is endemic is to avoid mosquito bites, particularly during the rainy season.

## References

[1] Gibbons RV, Vaughn DW. Dengue: an escalating problem. BMJ. 2002;324:1563–6. 10.1136/bmj.324.7353.1563 12089096 PMC1123504

[2] World Health Organization. Report of the meeting of the Scientific Working Group on Dengue. Geneva: The Organization: 2006. p. 1–168 [cited 2010 Jun 17]. http://apps.who.int/tdr/svc/publications/tdr-research-publications/swg-report-dengue

[3] Wichmann O, Gascon J, Schunk M, Puente S, Siikamaki H, Gjørup I, European Network on Surveillance of Imported Infectious Diseases. Severe dengue virus infection in travelers: risk factors and laboratory indicators. J Infect Dis. 2007;195:1089–96. 10.1086/512680 17357044

[4] World Health Organization. Dengue haemorrhagic fever: diagnosis, treatment, prevention and control. 2nd ed. Geneva: The Organization; 1997.

[5] Wichmann O, Muehlberger N, Jelinek T. Dengue—the underestimated risk in travellers. Dengue Bull. 2003;27:126–37.

[6] Vaughn DW, Green S, Kalayanarooj S, Innis BL, Nimmannitya S, Suntayakorn S, Dengue viremia titer, antibody response pattern, and virus serotype correlate with disease severity. J Infect Dis. 2000;181:2–9. 10.1086/315215 10608744

[7] Gubler DJ. The global emergence/resurgence of arboviral diseases as public health problems. Arch Med Res. 2002;33:330–42. 10.1016/S0188-4409(02)00378-8 12234522

[8] Jelinek T. Trends in the epidemiology of dengue fever and their relevance for importation to Europe. Euro Surveill. 2009;14:pii:19250. **PMID: 19555595** 19555595

[9] Wilson ME, Weld LH, Boggild A, Keystone JS, Kain KC, von Sonnenburg F, GeoSentinel Surveillance Network. Fever in returned travelers: results from the GeoSentinel Surveillance Network. Clin Infect Dis. 2007;44:1560–8. 10.1086/518173 17516399

[10] Cobelens FG, Groen J, Osterhaus AD, Leentvaar-Kuipers A, Wertheim-van Dillen PM, Kager PA. Incidence and risk factors of probable dengue virus infection among Dutch travellers to Asia. Trop Med Int Health. 2002;7:331–8. 10.1046/j.1365-3156.2002.00864.x 11952949

[11] Potasman I, Srugo I, Schwartz E. Dengue seroconversion among Israeli travelers to tropical countries. Emerg Infect Dis. 1999;5:824–7. 10.3201/eid0506.990615 10603220 PMC2640810

[12] Schwartz E, Weld LH, Wilder-Smith A, von Sonnenburg F, Keystone JS, Kain KC, GeoSentinel Surveillance Network. Seasonality, annual trends, and characteristics of dengue among ill returned travelers, 1997–2006. Emerg Infect Dis. 2008;14:1081–8. 10.3201/eid1407.071412 18598629 PMC2600332

[13] Schwartz E, Moskovitz A, Potasman I, Peri G, Grossman Z, Alkan ML. Changing epidemiology of dengue fever in travelers to Thailand. Eur J Clin Microbiol Infect Dis. 2000;19:784–6. 10.1007/s100960000388 11117644

[14] Informatics GIDEON. The Global Infectious Diseases and Epidemiology Online Network. 1994–2009 [cited 2009 Sep 29]. http://web.gideononline.com/web/epidemiology/

[15] Landelijk coördinatiecentrum reizigersadvisering. National Coordination Centre for Travelers’ Health Advice. National guidelines for travelers’ health advice. Amsterdam: Landelijk coördinatiecentrum reizigersadvisering; 2006.

[16] United Nations Economic and Social Development Agency. Definition of major areas and regions [cited 2009 Aug 6]. http://esa.un.org/unpp/definition.html

[17] Groen J, Koraka P, Velzing J, Copra C, Osterhaus AD. Evaluation of six immunoassays for detection of dengue virus-specific immunoglobulin M and G antibodies. Clin Diagn Lab Immunol. 2000;7:867–71. 11063489 10.1128/cdli.7.6.867-871.2000 PMC95976

[18] Lam SK, Devine PL. Evaluation of capture ELISA and rapid immunochromatographic test for the determination of IgM and IgG antibodies produced during dengue infection. Clin Diagn Virol. 1998;10:75–81. 10.1016/S0928-0197(98)00002-6 9646004

[19] Vaughn DW, Nisalak A, Solomon T, Kalayanarooj S, Nguyen MD, Kneen R, Rapid serologic diagnosis of dengue virus infection using a commercial capture ELISA that distinguishes primary and secondary infections. Am J Trop Med Hyg. 1999;60:693–8. 10348250 10.4269/ajtmh.1999.60.693

[20] McBride WJ, Mullner H, LaBrooy JT, Wronski I. The 1993 dengue 2 epidemic in North Queensland: a serosurvey and comparison of hemagglutination inhibition with an ELISA. Am J Trop Med Hyg. 1998;59:457–61. 9749644 10.4269/ajtmh.1998.59.457

[21] Balmaseda A, Saborio S, Tellez Y, Mercado JC, Pérez L, Hammond SN, Evaluation of immunological markers in serum, filter-paper blood spots, and saliva for dengue diagnosis and epidemiological studies. J Clin Virol. 2008;43:287–91. 10.1016/j.jcv.2008.07.016 18783984

[22] World Meteorological Organisation. World Weather Information Service [cited 2010 Jun 8]. http://www.worldweather.org/

[23] Freedman DO, Weld LH, Kozarsky PE, Fisk T, Robins R, von Sonnenburg F, GeoSentinel Surveillance Network. Spectrum of disease and relation to place of exposure among ill returned travelers. [Erratum in: N Engl J Med. 2006;355: 967]. N Engl J Med. 2006;354:119–30. 10.1056/NEJMoa051331 16407507

[24] Wilder-Smith A, Schwartz E. Dengue in travelers. N Engl J Med. 2005;353:924–32. 10.1056/NEJMra041927 16135837

[25] Mohammed HP, Ramos MM, Rivera A, Johansson M, Munoz-Jordan JL, Sun W, Travel-associated dengue infections in the United States, 1996 to 2005. J Travel Med. 2010;17:8–14. 10.1111/j.1708-8305.2009.00374.x 20074096

[26] Leder K, Wilson ME, Freedman DO, Torresi J. A comparative analysis of methodological approaches used for estimating risk in travel medicine. J Travel Med. 2008;15:263–72. 10.1111/j.1708-8305.2008.00218.x 18666927

[27] Franco L, Di Caro A, Carletti F, Vapalahti O, Renaudat C, Zeller H, Recent expansion of dengue virus serotype 3 in west Africa. Euro Surveill. 2010;15:pii: 19490. **PMID: 20184854** 20184854

[28] World Health Organization. Outbreak news. Chikungunya and dengue, south-west Indian Ocean. Wkly Epidemiol Rec. 2006;81:106–8. 16673456

[29] Van Herck K, Van Damme P, Castelli F, Zuckerman J, Nothdurft H, Dahlgren AL, Knowledge, attitudes and practices in travel-related infectious diseases: the European airport survey. J Travel Med. 2004;11:3–8. 10.2310/7060.2004.13609 14769280

[30] Massad E, Wilder-Smith A. Risk estimates of dengue in travellers to dengue endemic areas using mathematical models. J Travel Med. 2009;16:191–3. 10.1111/j.1708-8305.2009.00310.x 19538580

[31] Schwartz E, Mileguir F, Grossman Z, Mendelson E. Evaluation of ELISA-based sero-diagnosis of dengue fever in travelers. J Clin Virol. 2000;19:169–73. 10.1016/S1386-6532(00)00114-1 11090753

[32] Roehrig JT, Hombach J, Barrett AD. Guidelines for plaque-reduction neutralization testing of human antibodies to dengue viruses. Viral Immunol. 2008;21:123–32. 10.1089/vim.2008.0007 18476771

[33] Lanciotti RS. Molecular amplification assays for the detection of flaviviruses. Adv Virus Res. 2003;61:67–99. 10.1016/S0065-3527(03)61002-X 14714430

